# Page kidney following spontaneous subcapsular hematoma immediately after kidney transplantation: a case report

**DOI:** 10.1186/s12882-022-02855-y

**Published:** 2022-07-07

**Authors:** Tae Won Lee, Wooram Bae, Jungyoon Choi, Eunjin Bae, Ha Nee Jang, Se-Ho Chang, Dong Jun Park

**Affiliations:** 1 Department of Internal Medicine, Gyeongsang National University Changwon Hospital, Changwon, South Korea; 2 Department of Internal Medicine, Changwon Gyeongsang National University Hospital and Gyeongsang National University College of Medicine, 11 Samjungja-ro Sungsan-gu, Changwon, 51472 Republic of Korea; 3 Institute of Health Science, Gyeongsang National University, Jinju, South Korea; 4 Department of Internal Medicine, Gyeongsang National University Hospital, Jinju, South Korea

**Keywords:** Renal transplantation, Page kidney, Subcapsular hematoma, Allograft dysfunction

## Abstract

**Background:**

Page kidney (PK) is the occurrence of kidney hypoperfusion and ischemia due to pressure on the kidney by a subcapsular hematoma (SH), a mass, or fluid collection. SH after renal transplantation may result in kidney ischemia and graft loss.

**Case presentation:**

We present a rare case of early spontaneous SH in an allograft kidney that led to a decrease in renal function. A 56-year-old male patient underwent deceased donor kidney transplantation. After declamping, appropriate renal perfusion and immediate diuresis were observed, with no evidence of SH. However, his urinary output abruptly decreased 6 h postoperatively. Abdominal ultrasonography showed 28 mm deep SH on transplant and the resistive index (RI) increased to 0.98–1 and diastolic flow reversal was observed. Surgical interventions were performed 2 days after transplantation, following a further decrease in urinary output. Serum creatinine decreased to 2.2 mg/dL, urinary output increased to an average of 200 cc per hour and the RI value was decreased to 0.7 on POD 7.

**Conclusion:**

In patients with abrupt decreased renal function after transplantation, SH should be suspected and the presence of PK should be determined using Doppler USG. In these cases, surgical intervention may avoid allograft dysfunction.

## Background

Page kidney (PK), or Page phenomenon, is a form of treatable secondary hypertension that is due to external compression of the renal parenchyma, such as by subcapsular hematoma (SH), a mass, or fluid collection, leading to activation of the renin-angiotensin-aldosterone system [1–4]. SH-related PK is a challenging condition, as it may endanger kidney function or even constitute a life-threatening event, particularly in kidney-transplant recipients [5]. PK in renal transplants has been reported [3, 5–7], mainly following renal biopsy in the context of kidney transplantation [3]. The treatment of SH in renal transplant patients is controversial. In the absence of spontaneous resolution [8, 9], treatment options include antihypertensive agents, percutaneous drainage, surgical decortication, laparoscopic intervention, and nephrectomy [10–12]. We present the case of a patient with PK due to spontaneous SH that developed soon after renal transplant but was successfully managed by early surgical intervention.

## Case presentation

A 56-year-old male patient who had been on hemodialysis for 2 years for diabetic end-stage renal disease underwent deceased kidney transplantation in the right iliac fossa. Pre-transplantation induction treatment consisted of basiliximab, together with an immunosuppression regimen that included tacrolimus, mycophenolic acid, and methylprednisolone. No bleeding or other complications were observed during surgery. Anastomosis of the external iliac artery and vein and the renal artery and vein was performed and renal perfusion was started. JJ stent was not inserted. There was no bleeding in the anastomotic area and renal parenchymal blood flow was normal as viewed via Doppler ultrasonography (USG), performed in the operating room. After the anastomosis, immediate diuresis was observed. Surgery was completed without complications and the patient was admitted to the intensive care unit (ICU). Perdipine and fentanyl were infused intravenously for blood pressure (BP) and pain control, respectively.

**Fig. 1:**
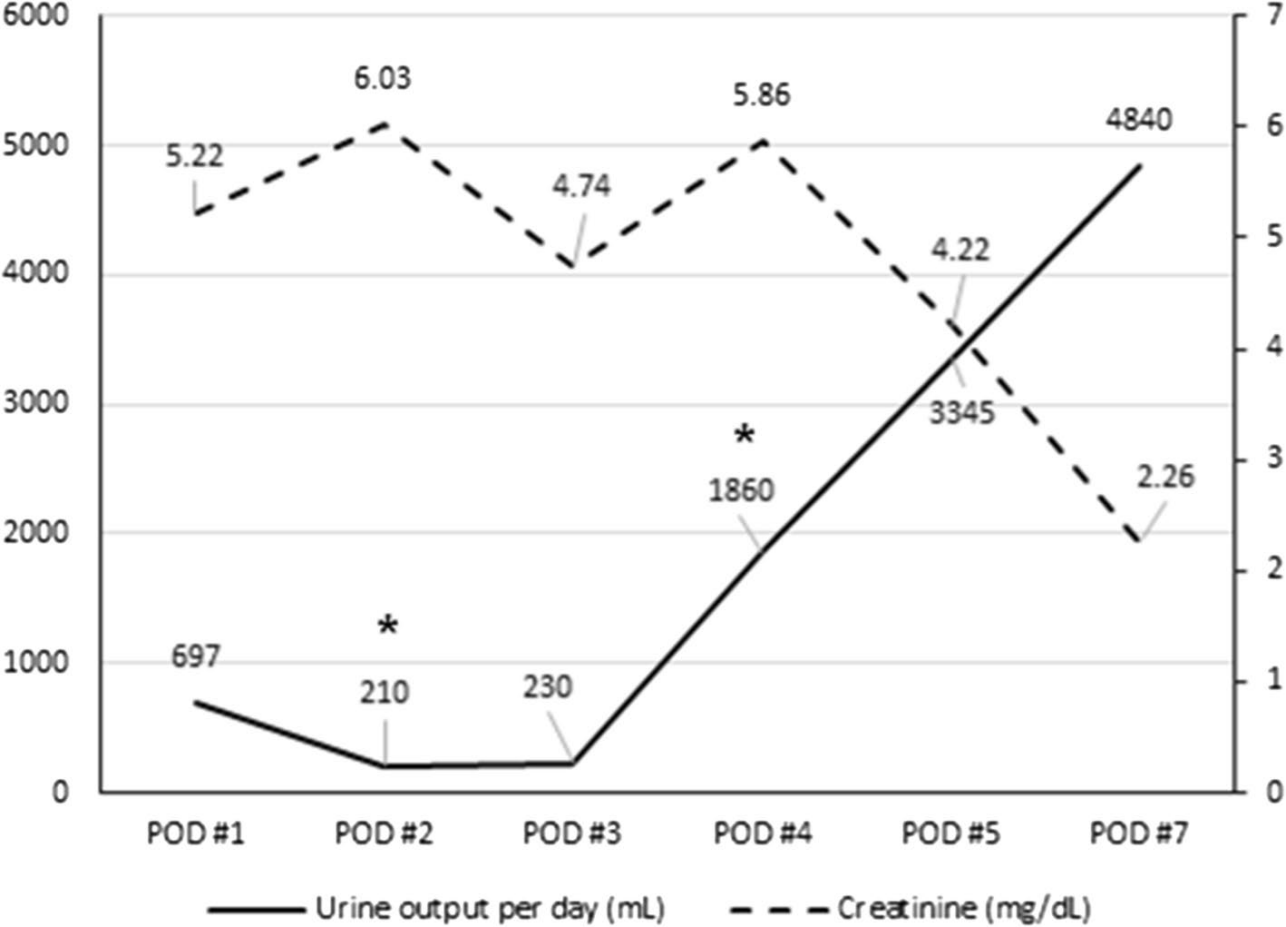
The change of urine output (solid line) and serum creatinine (dashed line) after transplantation. *Hemodialysis was done second and fourth day after transplantation

**Fig. 2:**
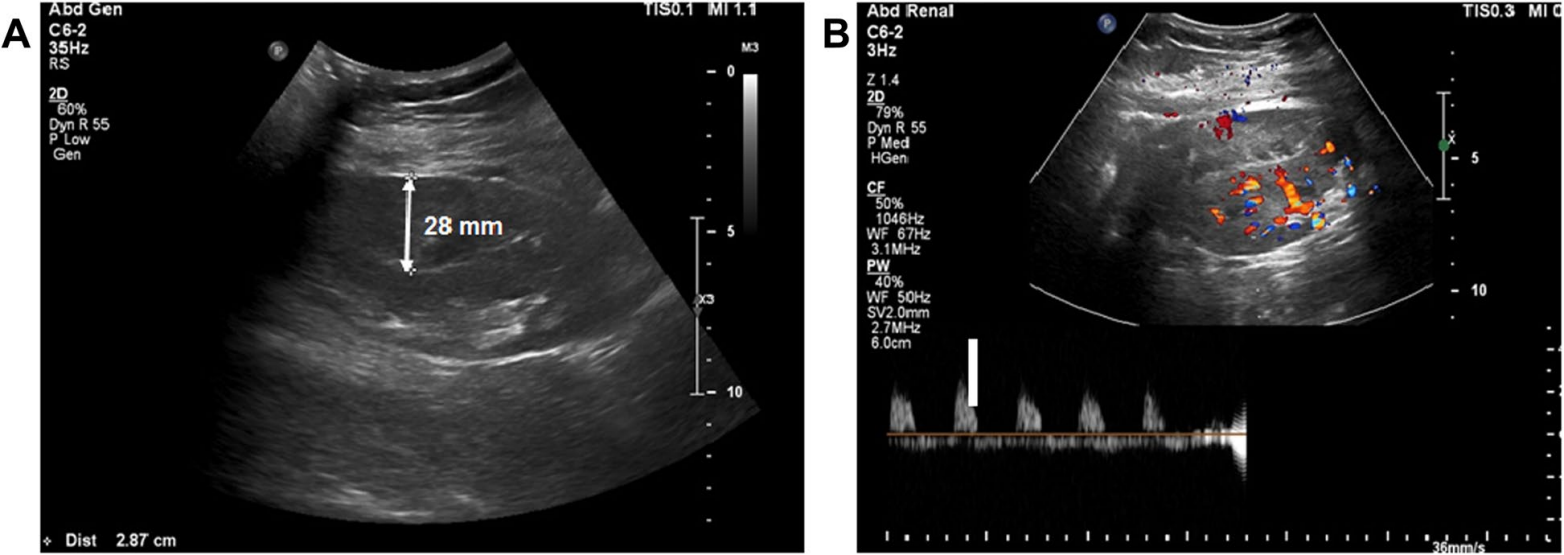
Doppler USG showing 28 mm in depth hematoma (**A**) and diastolic flow reversal (resistive index = 0.9–1) (**B**)

The patient’s urinary output was maintained at an average of 100 cc per hour. His initial vital signs were as follows: blood pressure, 145/85 mmHg; heart rate, 92 beats/min; respiratory rate, 20 breaths/min; and body temperature, 36.5 °C. They remained stable during the first 6 h after surgery. On postoperative day (POD) 1, His medication included immunosuppression such as tacrolimus, mycophenolic acid, and methylprednisolone. He did not take any anti-coagulant medications. The patient’s urine volume decreased to an average of 29 cc per hour. Saline infusion and diuretic treatment were performed to increase urinary output but no change in urine volume was observed. His serum creatinine level was 6.6 mg/dL preoperatively and 5.2 mg/dL on POD 1 (Fig. 1). No BP surge occurred on POD 1. Doppler USG showed no evidence of hydronephrosis but the renal cortex was compressed by a 28 mm deep SH (Fig. 2A). USG did not reveal evidence of rejection, renal vein thrombosis, or anastomotic stenosis in the graft artery or vein. The resistive index (RI) increased to 0.98–1 and diastolic flow reversal was observed (Fig. 2B).

**Fig. 3:**
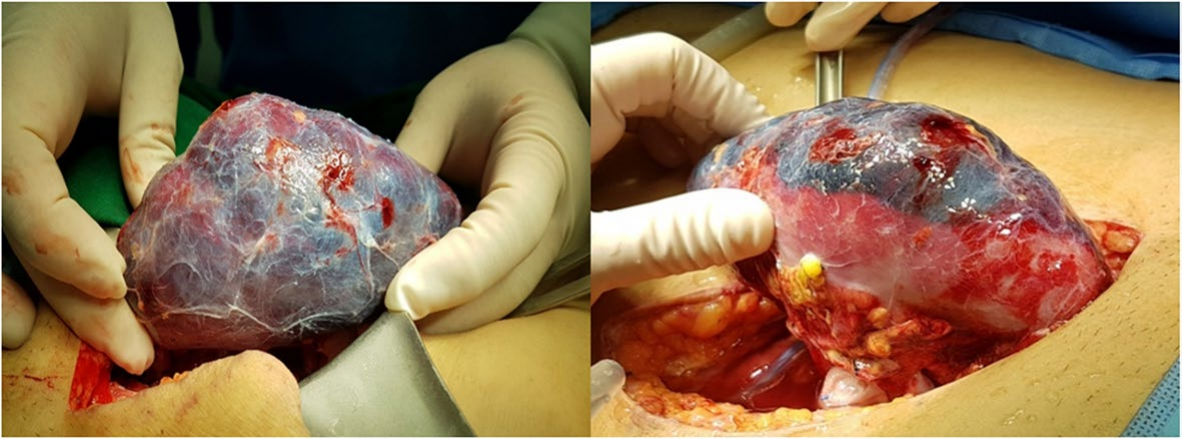
Subcapsular hematoma covering about 75% of the entire kidney surface area

On POD 2, the patient’s urinary output decreased further, to an average of 8.7 cc per hour, and subsequently to essentially zero. There was no change in the size of the SH or in the RI value in follow-up Doppler USG of the kidney. The serum creatinine level was 6.03 mg/dL. There was also no BP surge or allograft pain complaint. Hemodialysis was performed to relieve the volume overload. PK due to SH was considered to be the cause of the 6 h of continuous anuria. Emergency surgery was performed and SH covered about 75% of the entire kidney surface area was found (Fig. 3). Oozing was observed at several sites but there was no leakage of the arteriovenous or ureter anastomosis. A capsulotomy and hematoma removal was performed to alleviate the pressure on the renal parenchyma. Complete hematoma was not done due to extensive location across kidney surface. A biphasic sound was recorded via handheld vascular Doppler. There were no abnormalities in cortical blood flow. Additional hemostasis was performed at the oozing site. No immediate surgical complications developed. We were told that neither SH nor allograft dysfunction occurred in the recipient of the other kidney from the same deceased donor.

On POD 3, urine excretion decreased to an average of 9.6 cc per hour and the serum creatinine level was 4.7 mg/dL, attributed to the effects of hemodialysis. Additional hemodialysis was carried out for volume control and urinary output increased to an average of 78 cc per hour on POD 4. A decrease in creatinine level to 2.2 mg/dL and an increase in urinary output to an average of 200 cc per hour were recorded on POD 7 (Fig. 1). Doppler USG revealed that the hematoma had decreased to a depth of 18 mm and the RI value was 0.7. At discharge, the patient’s creatinine level was 0.98 mg/dL. He is currently being followed up in our outpatient department. His renal function has been maintained and he continues to take immunosuppressants.

## Discussion and conclusions

This report describes a case of PK by spontaneous SH, leading to acute kidney graft dysfunction, that occurred within 24 h of renal transplantation in a patient without a history of preceding trauma. The abrupt decrease in urinary output and the Doppler USG findings were the keys to the diagnosis. Early surgical intervention restored renal function.

The development of PK in an allograft patient is rare, with most cases induced by allograft biopsy, lymphocele, or trauma [1, 2, 12]. However, there have been two cases of PK that occurred early after renal transplantation in the absence of any of the above causes [7, 12]. Iovino et al. described an aggravation of graft dysfunction, with SH confirmed by Doppler USG on POD 13. Surgical intervention, including hematoma evacuation, resulted in functional recovery of the allograft. The cause of the SH was not noted. Hori et al. reported allograft dysfunction triggered by PK due to SH, which was not detected by routine USG but was instead discovered during surgical exploration [12]. In their patient, rupture of a small renal cyst was the likely cause of the SH. Allograft function was restored after capsulotomy and hematoma evacuation. Our case was unique in that PK occurred spontaneously, without any detectable lesion, early after transplantation.

Acute pain over the allograft, uncontrolled hypertension despite appropriate medicines, and a reduction of urinary output are useful signs in the diagnosis of early stage PK [1]. Other important findings are SH and an elevated RI on Doppler USG [1, 12]. Our patient could not sense graft pain as he received a continuous intravenous infusion of narcotic analgesics immediately after allograft surgery. Hypertension was well-controlled with intravenous anti-hypertensive medicines. PK was suspected based on the sudden decrease in urinary output between 6 h and 48 h post-transplantation, the absence of a decrease in the serum creatinine level, and the detection of a 28 mm deep SH via Doppler USG, routinely performed in renal transplant patients on POD 1. Although when the subcapsular bleeding started was unknown, a hemodynamic change was not apparent until 6 h after surgery.

Marginal grafts are increasingly being used in transplant recipients due to the high demand for kidney transplants, and the risk for primary non-function, delayed graft function, or acute post-surgical complications, such as capsular detachment and subsequent hematoma, are accordingly higher [7]. To address these risks, diagnostic renal biopsy is often performed, although it is the most common cause of sub-capsular bleeding [1] and surgical complications, including SH, thus increasing the risk for PK. Accordingly, PK should be considered in marginal-graft recipients with an abrupt contraction of diuresis, an increase in serum creatinine, and delayed graft function. Our patient did not receive a marginal graft, although his kidney was from a deceased donor. As SH did not occur in the recipient of the other kidney from the same deceased donor, a systemic component, such as coagulopathy, in the donor could be ruled out as the cause of the surgical complications.

Renal transplant recipients benefit from follow-up imaging and monitoring strategies, given the complexity of the surgical procedure and the need for an ICU stay for a few days after transplantation. USG is the principal imaging modality in the evaluation of renal transplants, analogous to its importance in detecting vascular pathologies. It can be easily performed at the bedside, detects possible complications, and guides further imaging or intervention [13]. Doppler USG is also used in renal biopsy, the drainage of fluid collection [14, 15], and to detect SH in allografts [5–9] although small hematomas may be missed [12]. Measurement of the RI in the case of presence of SH might provide valuable clues suggesting a diagnosis of PK [1, 7] and in our patient led to the restoration of renal function through early surgical treatment.

Allograft dysfunction induced by PK can be reduced by appropriate management before irreversible damage occurs [16], although the optimal intervention is a matter of debate [5, 12, 16]. Some clinicians have advocated a cautious wait-and-see approach in SH patients because of the possibility of spontaneous resolution [10, 11]. However, allograft dysfunction due to PK in a transplanted kidney may occur if the diagnosis and subsequent intervention are delayed [12]. In such cases, even partial ischemia for a prolonged period of time might result in irreversible damage, a scenario that can be avoided by early intervention. Our case was unique in that PK immediately occurred after transplantation compared to other cases that usually appear late. This naturally reminds us of the issue of the early diagnosis and decision for management. Our patient was diagnosed relatively quickly, allowing timely surgery and the complete restoration of allograft function. This experience demonstrates the importance of an early diagnosis and early surgical intervention in patients with decreased renal function due to PK in a transplanted kidney.

In conclusion, PK should be considered a cause of allograft dysfunction in the early period after transplantation. Early and accurate diagnosis followed by appropriate surgical intervention can avoid partial or permanent renal damage.

## Data Availability

The data supporting the conclusions of this article is included within the article.

## Abbreviations

BP: Blood pressure
ICU: Intensive care unit
PK: Page kidney
POD: Postoperative day
RI: Resistive index
SH: Subcapsular hematoma
USG: Ultrasonography
